# Occurrence of *Campylobacter* spp. in Selected Small Scale Commercial Broiler Farms of Bangladesh Related to Good Farm Practices

**DOI:** 10.3390/microorganisms8111778

**Published:** 2020-11-13

**Authors:** Badrul Alam, Md. Nasir Uddin, Debashish Mridha, A. H. M. Taslima Akhter, SK Shaheenur Islam, A. K. M. Ziaul Haque, S. M. Lutful Kabir

**Affiliations:** 1 Department of Microbiology and Hygiene, Bangladesh Agricultural University, Mymensingh 2202, Bangladesh; badrul.alamjstu@gmail.com (B.A.); nasirmbsobug@gmail.com (M.N.U.); debashish.dip20@gmail.com (D.M.); vetzia.2004.bd@gmail.com (A.K.M.Z.H.); 2 FAO-Food Safety Program (FSP), Institute of Public Health, Mohakhali, Dhaka 1215, Bangladesh; takhter36@yahoo.com; 3 Department of Livestock Services, Krishi Khamar Sarak, Farmgate, Dhaka 1215, Bangladesh; s_islam73@live.com

**Keywords:** *Campylobacter* spp., occurrence, broiler farms, molecular detection, multidrug resistant, Bangladesh

## Abstract

Poultry origin *Campylobacter* is considered as one of the leading causal agents of human foodborne illness. This study was conducted to estimate the occurrence, molecular identification, and antimicrobial resistance (AMR) of *Campylobacter* species from the broiler farms in Bangladesh. Samples (352) were collected from 32 farms and comprised of 128 cloacal swab, 64 feed, 64 drinking water, 64 attendants’ hand rinsed water, and 32 whole carcasses. All samples were tested for the presence of *Campylobacter* via cultural, biochemical, and PCR. The AMR was determined via the disc diffusion method. An overall occurrence of *Campylobacter* spp. was estimated as 26.4%. The level of *Campylobacter* contamination was found to be higher in conventional farms (36.4%) than the good practice farms (16.5%) including all sample categories (*p* = 0.000). Of 93 isolates, 67.74% and 32.26% were confirmed as *C. jejuni* and *C. coli* respectively, of which 34.92% *C. jejuni*, and 30% *C. coli* were shown to be multidrug-resistant. A significant occurrence of *Campylobacter* contamination in broiler farms with multidrug resistant patterns might be cogitated as serious food safety and public health concern linking to poultry food chain. A risk reduction approach through good farming practices targeting the prudent use of antimicrobials for broiler production is thus necessitated.

## 1. Introduction

*Campylobacter* spp. are food-borne pathogens which are microaerophilic, spiral, or slightly curved, motile, non-spore forming Gram-negative bacteria having a single flagellum at one or both poles [1]. The thermophilic *Campylobacter* is well documented in poultry mostly in broilers and turkeys [2]. However, this organism is infrequently identified in commercial broiler flocks under the age of 2–3 weeks [3]. *Campylobacter* is recognized as one of the leading causal agents of foodborne gastroenteritis in humans. Every year a large number of people are affected by this pathogen globally [4]. A total of 246,307 human cases were confirmed as *Campylobacter* infection by the European Food Safety Authority (EFSA) as the highest occurrence for any bacterial pathogen in Europe, of which *C. jejuni* and *C. coli* were accountable as 83.6% and 8.5% respectively [5].

Among the *Campylobacter* spp., *C. jejuni*, *C. coli*, and *C. lari* can transmit from the intestinal tract of animals and may be contaminated animal origin foods. Amongst the species of *Campylobacter*, *C. jejuni* and *C. coli* are regarded to be a major contributor (around 90%) of human campylobacteriosis [6]. Since these species of *Campylobacter* were documented as causal agents of human diarrheal disease, their presence in food indicate a potential health hazard. Apart from the enteric diseases, *Campylobacter* infection can cause Guillain–Barré syndrome (GBS), with severe chronic symptoms following the infection, and characterized by acute paralysis resulting from an autoimmune and slow healing rate [7]. In poultry supply chain, the main phases of contamination namely farm response, transport, slaughtering, and meat processing in the live bird markets (LBMs) and consumption. These pathogens can remain viable along the food chains with high accumulation at the consumer level [8]. 

Amongst the *Campylobacter* isolates, *C. jejuni* (51%) and *C. coli* (35.5%) are the more prevalent species in broiler meat [9] and transmission occurs due to the improper cleaning and disinfection of equipment and poultry crates [10]. Since various antimicrobial supplementations in poultry feed have linked with the development of AMR bacteria present in carcass and chicken meat products is now a paramount public health concern [11,12,13]. This practice has a high impact on food safety both in veterinary and human health, and considered to be devastating more rapidly in low- and middle- income countries (LMICs), where the imprudent use of antibiotics is more common to intensify livestock productivity [14]. Strict biosecurity measurement through the adoption good agriculture practices (GAP) in poultry farming could minimize the risk of introduction and later subsistence of these pathogens in commercial poultry flocks [15,16]. 

In Bangladesh, the major proportion (50–60%) of poultry meat comes from sector three small scale poultry production system, where a low to minimum level of biosecurity is being maintained at the rural settings [17], that facilitates to increase likelihood of *Campylobacter* spp. colonization in poultry. A significant number of studies have been conducted regarding the prevalence, molecular detection, and antibiotic sensitivity test of *Campylobacter* spp. in broiler and layer poultry in organized farms and LBMs of Bangladesh [18,19,20,21]. However, campylobacteriosis in the major production system (small scale sector three category) in terms of occurrence and its comparison with good farm practices has yet to be explored. 

Given the situation, it was pertinent to conduct this study that has confirmed (i) occurrence, (ii) molecular detection, and (iii) antimicrobial resistance pattern of *Campylobacter* spp. in broiler farms of rural Bangladesh. Further, the study has evaluated the *Campylobacter* positivity status among the two groups of farms such as good practice and conventional farms with an aim to decide the risk reduction options considering public health hazards. It is expected that this study will inform national polices on good poultry farming practices in the major production system considering emerging public threats and food safety grounds in low resource settings like Bangladesh. 

## 2. Materials and Methods 

### 2.1. Ethics Statement 

The Ethical Committee of the Bangladesh Agricultural University, Mymensingh approved the study on 19 August 2020 under reference no. AWEEC/BAU/2020(26). The farms were selected after consultation with the sub-district (Upazila) livestock office of Department of Livestock Services, Bangladesh, and with the willingness of the farmers to participate in this study. Thus, a written consent was taken from each of the farm owner/managers during the collection of samples.

### 2.2. Study Design and Location

**Figure 1:**
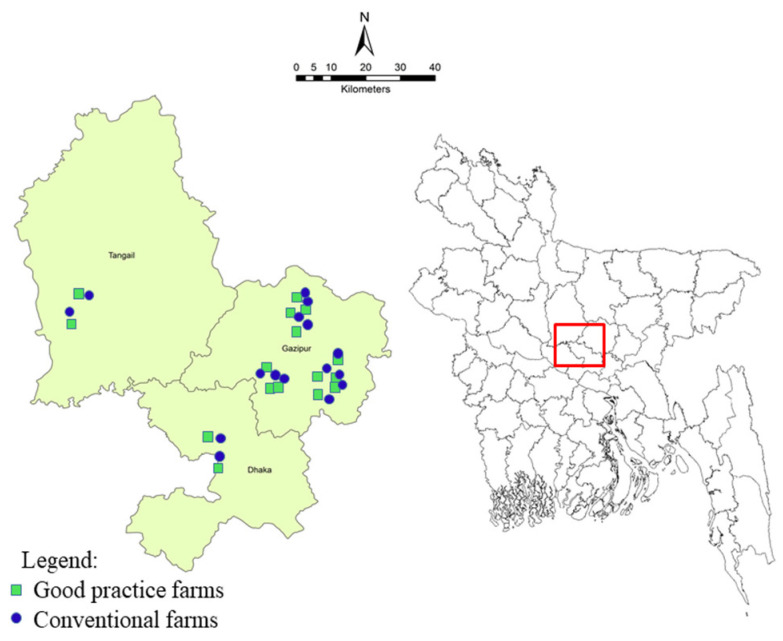
Location of broiler farms in three district of Bangladesh were included in this survey. Same number of farms were included in this study comprised of good practice broiler farms (*n* = 16) and conventional farms (*n* = 16) (4 farms from Dhaka, 4 from Tangail and 24 from Gazipur districts).

The cross-sectional survey was conducted in three poultry dominant districts (Dhaka, Gazipur and Tangail) of Bangladesh. The study utilized 32 broiler farms that comprised of 24 farms from Gazipur, 4 farms from Tangail and the remaining 4 farms from Dhaka districts were included under this survey after consultation with local livestock offices. The geospatial locations of the surveyed farms are presented in Figure 1. On the basis of density distribution of the broiler farms, more farms were enrolled from Gazipur district under this study.

### 2.3. Selection of Farms 

Of 32 farms, an equal number of farms (good practice farms, *n* = 16 and conventional farms, *n* = 16) were included in this study with an inclusion criterion of a minimum flock size of ≥1000 birds per farm under the sector three (small scale commercial) poultry production system. 

#### Development of Good Practice Farm

The good practice broiler farms were developed through a collaborative program of Food and Agriculture Organization (FAO) of the United Nations and Department of Livestock Services (DLS), Bangladesh in poultry food safety where five key biological control measures (bacteria) for safe broiler production were addressed at the farm level. The key control measures (good agriculture practices) relating to germs were implemented at the farms’ level, are presented in Table 1. 

Additionally, the good practices farms were supported through providing intervention materials for improving farm biosecurity and a day-long participatory training focusing on key control measures on safe broiler production. The impact of the interventions was monitored periodically by a local committee of each sub-district for assessment of effectiveness of these activities. To evaluate the *Campylobacter* positivity status among the two groups of farms, an equal number of conventional farms (*n* = 16) were taken randomly to match with the good practice farms from the same locations (Figure 1 and Supplementary Table S1 appendix).

### 2.4. Sample Collection and Processing 

An equal number of samples were randomly collected via a convenient sampling technique before the selling of birds (35–45 day of a production cycle) from both categories of farms spanning from August to October 2017. A sample collection checklist was used for recording all samples collected from each farm (Supplementary Table S2). Of 352 different types sample, 36.36% (*n* = 128) cloacal swab, 18.18% (*n* = 64) feed, 18.18% (*n* = 64) drinking water, 18.18% (*n* = 64) attendants’ hand rinse water, and 9.09% (*n* = 32) whole carcasses were collected after slaughtering in poultry stalls of LBMs. Aseptic measures were maintained during collection of samples, the amount of which varied as per sample category as 100 g for meat (carcass) and feed, 500 mL for water and 1–5 mL or mg cloacal swabbed materials in sterile cotton swab, and 200 mL attendants’ hand rinse water. The cotton swabs were preserved and transported in Cary Blair transport media. However, 32 whole carcass samples each represented a combination of breast, thigh, and drumstick. To overcome the sampling bias, three sub-samples of each sample category were randomly collected and pooled together from each farm. Immediate after collection, samples were kept in a plastic container and shifted to the laboratory of Department of Microbiology and Hygiene, Bangladesh Agricultural University (BAU), Mymensingh maintaining cool chain at 4–6 °C in an insulated foam box. The broiler farmers were informed duly on the research objectives so as to they could voluntarily involve with this study through arrangement of meetings in each sub-district livestock offices (*n* = 5) of three districts (Dhaka (1), Tangail (1) and Gazipur (3)). 

### 2.5. Isolation, Identification and Molecular Detection 

#### 2.5.1. Culture and Biochemical Tests

The samples were processed immediately after arrival in the laboratory for bacteriological culture. Isolation of *Campylobacter* spp. was carried out as previously described [22]. In briefly, 100 µL of homogenates of processed sample spread on the 0.45 µm cellulose nitrate filter paper (Sartorius Stedim Biotech, Göttingen, Germany) that were placed on the surface of Blood base agar no. 2 (BBA) plate (HiMedia, Mumbai India) supplemented with 5% sheep blood and allowed to stand for 30 min at room temperature. The filter was removed, and the medium plate was incubated at 37 °C for 48 h under microaerophilic conditions (5% O_2_, 10% CO_2_, and 85% N_2_) using AnaeroPouch^®^ MicroAero (Mitsubishi Gas Chemical Co., Inc., Tokyo, Japan). In BBA, grey, flat and irregularly spreading colonies were observed. The colonies from the selected media then sub-cultured onto the Blood agar base no. 2 with Skirrow supplement/growth supplement for getting a single and pure colony. To identify and differentiate between *C. jejuni* and *C. coli*, Gram staining and biochemical tests such as the catalase test, oxidase test, hippurate hydrolysis test, and motility test, were accomplished as earlier described [23,24]. Pure isolates were obtained in above techniques used for molecular assay.

#### 2.5.2. Molecular Detection

In PCR assay, DNA was extracted from the pure culture of *Campylobacter* spp. via the boiling method [25]. For confirmation of the genus of *Campylobacter*, 16S rRNA gene-based PCR was done [26]. After confirmation of *Campylobacter* spp., hippuricase (*hipO*) gene-based PCR was accomplished for the identification of *C. jejuni* [27]. Those isolates presented as negative interpretation via *hipO* gene-based PCR were further detected as *C. coli* through *cdtA* gene-based multiplex PCR assay as previously described [28]. The list of primers with thermal condition used in PCR are shown in Table 2. In briefly, for each isolate, 25 µL of reaction mixture was prepared by adding 12 µL master mixtures (Promega, Madison, WI, USA), 1 µL forward primer (10 pmol), 1 µL reverse primer (10 pmol) (BioServe Biotechnologies, Hyderabad, India), 3 µL DNA template, and rest portion taken as deionized water in a PCR tube for amplification. The PCR reactions were carried out using a thermocycler (Astec, Fukuoka, Japan) as per manufacturer’s instruction: initial denaturation with 1 cycle of 5 min at 94 °C, 30 cycles each consisting of denaturation as shown in Table 2 and a final extension step of 10 min at 72 °C. PCR products were visualized to gel electrophoresis (1.5–2% agarose, Invitrogen, Carlsbad, CA, USA) and stained with ethidium bromide (0.5 µg mL^−1^) and de-stained with distilled water, each for 10 min, before gel images were captured using a UV transilluminator (Biometra, Göttingen, Germany).

### 2.6. Antimicrobial Susceptibility Test

All *Campylobacter* spp. strains were tested against eight antimicrobial agents in Bangladesh, viz. amoxycillin (30 µg), azithromycin (30 µg), ciprofloxacin (5 µg), erythromycin (30 µg), gentamicin (10 µg), tetracycline (30 µg), streptomycin (10 µg) and norfloxacin (10 µg) (HiMedia, Mumbai, India) by disk diffusion method [29]. The zones of growth inhibition were related with the zone diameter interpretative standards as labeled by the Clinical and Laboratory Standard Institute [30], and thus inferred i.e., susceptible (S), intermediate resistant (I) or resistant (R) to the antimicrobial agents. *E. coli* strain ATCC 25922 was used as a quality control organism. All interpretations were verified by performing a minimum of two replicates of the disk diffusion assay. 

### 2.7. Data Management and Statistical Evaluation

The data for laboratory test were taken and recorded in Microsoft Excel^®^ worksheet and imported into Epi Info 7 program [31] for statistical analysis. The categorical data were presented as proportion (%). Chi-square tests were performed to assess the correlation of positivity status among the various locations (sub-district and district) and sample categories (cloacal swab, feed, water, attendants’ hand rinse water, and whole carcass) with good practice broiler farms versus conventional farms. Thereby, *p* ≤ 0.05 was used to determine statistical significance. 

## 3. Results

### 3.1. Occurrence of Campylobacter spp.

The survey confirmed an overall occurrence of *Campylobacter* spp. as 26.4% (93/352) through culture-based methods and biochemical tests. Further, a single representative colony of *Campylobacter* was isolated as a pure culture from each of 93 positive samples. A genus specific 16S rRNA PCR assay was used for generating an expected 1530 bp amplicon size, and validating the identity as *Campylobacter* spp. Among the three districts, Tangail was recorded as highest prevalence (29.5%) of *Campylobacter* spp. followed by Gazipur (26.5%) and Dhaka districts (22.7%). Relating to the district level positivity status, there was no significant difference observed (Table 3) and sub-district-wise contamination of *Campylobacter* spp., Kapasia was found to be highest degree of contamination (39.8%) followed by Sadar Tangail (29.6%) and Savar (22.7%) sub-districts. However, Sreepur and Sadar, Gazipur sub-districts were captured at lower levels of *Campylobacter* spp. infection (19.3% and 20.4% respectively). The studied sub-districts were found to be significant with *Campylobacter* infection (*p* = 0.014) in this study (Table 3). 

Among the two groups of farms, the overall occurrence of *Campylobacter* spp. was estimated as 36.4% and 16.5% in conventional farms and good practice farms respectively. Of collected different samples, the contamination of *Campylobacter* spp. in whole carcass was recorded as 56.3% (nine in 16) and 18.8% (three in 16) in conventional farms and good practice farms respectively. Similarly, 48.4% (31 in 64), 20.3% (13 in 64) in cloacal swab, 25% (eight in 32), and 15.63% (five in 32) in feed, 25% (eight in 32) and 12.5% (four in 32) in attendants’ hand rinse water, and 25% (eight in 32) and 12.5% (four in 32) in drinking water were captured in conventional and good practice broiler farms respectively. Considering the level of contamination, the conventional farms were found to demonstrate higher occurrence of *Campylobacter* spp. than the good practice farms, including all categories of sample and found to be statistically significant (Table 4).

### 3.2. Molecular Detection by Polymerase Chain Reaction (PCR)

All *Campylobacter* isolates presented specific amplification (1530 bp) through genus specific (16S rRNA gene) polymerase chain reaction (PCR). However, *hipO* gene-based PCR was completed to identify *C. jejuni* and all *C. jejuni* isolates presented specific amplification (735 bp). In addition, *cdtA* gene-based multiplex PCR was conducted for the confirmation of *C. coli* and obtained specific amplification (329 bp) (Supplementary Figure S1). 

**Figure 2:**
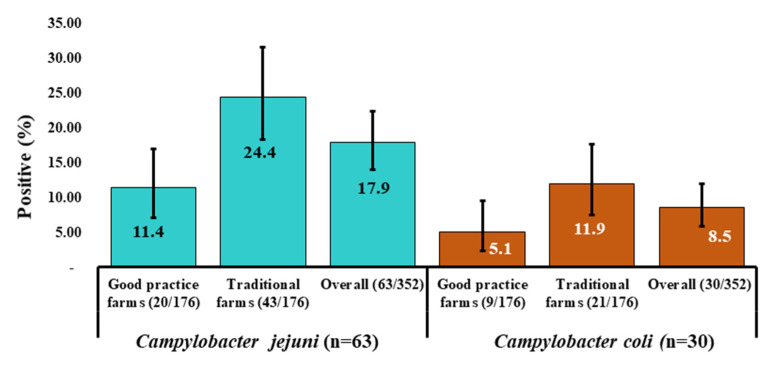
Distribution of occurrence of *Campylobacter* isolates (95% Confidence interval) that included *C. jejuni* (*n* = 63) and *C. coli* (*n* = 30) in two groups farms (good practice and traditional farms) in three districts of Bangladesh. The overall occurrence of *Campylobacter* spp. among the groups were significantly different (*p* = 0.000).

Of the 93 isolates, 67.7% (*n* = 63) as *C. jejuni* and remaining 32.3% (*n* = 30) as *C. coli* were confirmed through the molecular detection. In good practice farms, 16.5% (29 in 176) of samples were found to be positive with *Campylobacter* that showed *C. jejuni* and *C. coli* occurrence as 11.4% (20 in 176) and 5.1% (nine in 176) respectively (Figure 2). In addition, in 36.4% (64 in 176) samples from conventional farms were confirmed with *Campylobacter* infection, where *C. jejuni* and *C. coli* positive status was 24.4% (43 in 176) and 11.9% (21 in 176) respectively (Figure 2). However, the overall occurrence of *C. jejuni* and *C. coli* was confirmed as 17.9% (63 in 352) and 8.5% (30 in 352), respectively (Figure 2).

### 3.3. Antimicrobial Susceptibility Test

**Figure 3:**
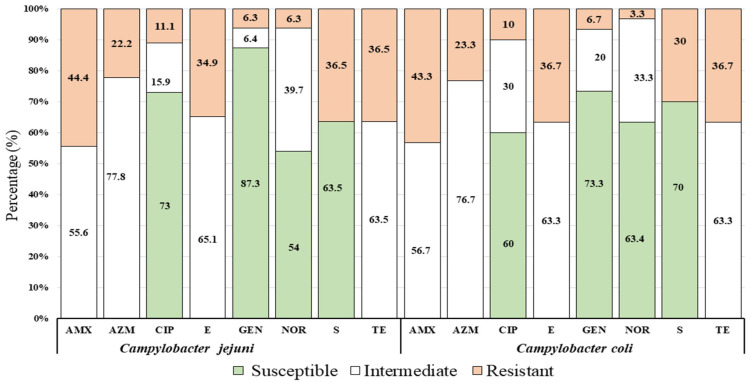
Proportion of antimicrobial susceptibility profile of *Campylobacter* spp. (*C. jejuni* and *C. coli*) against common eight (8) antimicrobial agents with standard doses (μg): Amoxicillin (AMX, 30 µg), Azithromycin (AZM, 30 µg), Ciprofloxacin (CIP, 5 µg), Erythromycin (E, 30 µg), Gentamycin (GEN, 10 µg), Norfloxacin (NOR, 10 µg), Streptomycin (S, 10 µg), and Tetracycline (TE, 30 µg) showed in three groups (susceptible, intermediate, and resistant) of the resistant pattern in accordance to Clinical and Laboratory Standards Institute [30].

Of 63 isolates of *C. jejuni*, 73% (*n* = 56), 87.3% (*n* = 55), 54 % (*n* = 34), and 63.5% (*n* = 40) were found to be susceptible to ciprofloxacin, gentamicin, norfloxacin, and streptomycin, respectively. In contrast, out of 30 isolates of *C. coli*, 60 % (*n* = 18), 73.3% (*n* = 22), 63.4 % (*n* = 19), and 70% (*n* = 21) were captured as to be susceptible to ciprofloxacin, gentamicin, norfloxacin, and streptomycin, respectively (Figure 3).

### 3.4. Antimicrobial Resistance Pattern

Out of 63 isolates of *C. jejuni*, 34.9% (*n* = 22) were revealed resistant against three or more antimicrobial agents as multidrug resistant namely amoxicillin, streptomycin, tetracycline, erythromycin, ciprofloxacin, amoxicillin, norfloxacin, and azithromycin. Of 22 isolates, 19% (*n* = 12) were presented as resistant against three antimicrobials as AMX-S-TE (7.9%, *n* = 5) and E-S-CIP (11.1%, *n* = 7), respectively, and 15.9% (*n* = 10) were shown to be multidrug resistant against four antimicrobial agents (AMX-NOR-AZM-TE). Conversely, 14.3% (*n* = 9) isolates of *C. jejuni*, were shown to be resistant against two antimicrobials, namely AMX-TE (6.4%, *n* = 4) and AMX-S (7.9%, *n* = 5), respectively, and 50.8% (*n* = 32) of isolates were shown to be resistant against a single antimicrobial agent (Table 5). 

Out of 30 isolates, *C. coli*, 30% (*n* = 9) were captured as multidrug resistant. Of 9 isolates, 16.7% (*n* = 5) isolates were presented resistant against three antimicrobials viz. AMX-S-TE (6.7%, *n* = 2) and E-S-CIP (10%, *n* = 3) respectively, and 13.3% (*n* = 4) were shown as multidrug resistant against four antimicrobials (AMX-NOR-AZM-TE). In this study 13.3% (*n* = 4) isolates were shown to be resistant against two antimicrobials, namely AMX-TE (6.7%, *n* = 2) and AMX-S (6.7%, *n* = 2), respectively, and 56.7% (*n* = 17) isolates were presented as resistant simply to a single antimicrobial agent (Table 5).

## 4. Discussion

The study was conducted in three major poultry districts of Bangladesh neighboring the capital city and these districts provide chicken meat and eggs for the consumption of city dwellers. Bangladesh is now self-reliant in meat production, of which the maximum contribution comes from broiler meat as the government has taken various measures to support the country’s livestock sector [32]. A lack of substantial data prompts the difficulty of understanding the current status of poultry diseases [33]. Among the different types of poultry diseases, both bacterial and viral origin are the major burden in poultry sectors, of which campylobacteriosis and salmonellosis are most prevalent [34]. Therefore, this survey was sensible to estimate the distribution of *Campylobacter* spp., in major broiler production farms that will support to the national disease control program as a baseline data.

We estimated an overall positive status of *Campylobacter* spp. as 26.4% (95% CI: 21.9–31.3%) in boiler farms. The overall positivity status estimated under this study has been supported by other research both in home and abroad. A positive status of 32% *Campylobacter* in broiler flocks was found in India [35], and 29% and 21.5% in Pakistan [36,37]. However, *Campylobacter* occurrence in broiler samples was confirmed as 32% and 40.5% in Bangladesh [20,21]. Conversely, relatively a higher prevalence of *Campylobacter* in broiler samples was reported in Sri Lanka as 67% as a result of higher temperature in this country comparing to the other parts of Indian subcontinent [38]. In Europe, the mean prevalence of *Campylobacter* in colonized broiler and carcasses was reported as significantly higher than others as 71.2% and 75.8% respectively [9]. Moreover, *Campylobacter* colonization was stated as approximately 90% in broiler flocks in the USA [39] and 65% in Poland [40].

In this survey, the degree of higher contamination was documented in whole carcass (56.3%). This finding has been confirmed closely by many studies in Asian counties, *Campylobacter* positivity was documented as 59% Pakistan and 40% in Sri Lanka in broiler meat samples respectively [37,38]. Moreover, *Campylobacter* positivity was established in broiler carcasses samples as 45.1% in China [41], 31% in Thailand [42], 30% in Vietnam [43], and 80.9% in Cambodia [44]. However, noticeably, a higher prevalence (75.8%) of *Campylobacter* spp. was documented in broiler carcass of European countries [9]. 

The drinking water of 18.8% (12/64) poultry farms was found to be contaminated with *Campylobacter*. The result obtained from this study was corroborated by another research conducted in Bangladesh as presence of 33% *Campylobacter* contamination in drinking water samples from broiler farms [21]. The present study further confirmed as 18.8% (12/64) farm attendants’ hand rinsed water were found to be *Campylobacter* positive as they were exposed to contaminated flocks and water or even poultry cages during working at the farms. The *Campylobacter* can survive in hands at a log CFU loss in 45 min [45] and human moist clothing 0.5–24 h at room temperature [46]. The likelihood of enteric infections among poultry attendants is enormous as they become exposed to *Campylobacter* contamination. Therefore, personal hygienic measures to be taken immediately after working at poultry farms. However, use of protective materials like mask and gloves, aprons are needed that will minimize further exposure of zoonotic pathogens [47,48]. Likewise, the study confirmed 20.3% (13 in 64) feed samples found to be positive with *Campylobacter* that disagrees with the other research as 0% *Campylobacter* positive status confirmed [21]. Since the feed sampling was accomplished using the pool samples (both from feeder and stored feed) from the surveyed farms, this positivity status of the feed sample due to cross contamination from the infected feeds at poultry feeder. 

The study has enumerated as higher occurrence of *Campylobacter* spp. in conventional farms (36.4%, 95% CI: 29.3–43.9%) in comparison to the good practice farms (16.5%, 95% CI: 11.3–22.8%). This finding is empirically supported by other research [49] as high standards of biosecurity measures will reduce the *Campylobacter* contamination by 20–40% lower than those farms with lower standards. Moreover, biosecurity measurement is important targeted to *Campylobacter* control when colonization happens in a poultry flocks the horizontal spread can be prompt [50].The likelihood of bacterial infection was lower in best practice farms and found to be more protective than the poor practice farms because of the implementation of key control measures related to farm biosecurity and GAP practices, i.e., provision of perimeter fencing, netting of the farm to control entrance of wild and domestic animals and birds, controlling human movement inside the farm, dedicated footwear, and footwear cleaning at the entry to the poultry shed with disinfectants, all in all out practices, along with use of safe production inputs (DOC, feed, and water) [15,51,52,53]. In this study, the overall occurrence of *C. jejuni* (17.9%, 63 in 352) was found to be higher than *C. coli* (8.5%, 30 in 352). Among the isolates, 67.74% *C. jejuni* and 32.26% *C. coli* were confirmed through gene specific PCR assays. This observation was consistent with the many studies [21,36,37,54,55]. However, a higher occurrence of 51% *C. jejuni* and 35.5% *C. coli* were detected in broiler meat in Europe [9]. In contrast, some researchers reported *C. coli* is the more abundance species in broiler poultry [38,56].

This study recognized the antimicrobial resistance patterns of *C. jejuni* and *C. coli* as 44.4% and 43.3% to amoxicillin, 36.5% and 36.7% to tetracycline, and 34.9% and 36.7% to erythromycin, respectively. Similar observations have been proven by many researchers in Bangladesh [13,18,19]. However, a dissonant finding was confirmed as *C. jejuni* and *C. coli* are resistant to 5%, 11.1% to erythromycin and 85%, 24.4% to tetracycline, respectively [38]. 

In this study, 34.9% (*n* = 22) *C. jejuni* and 30% (*n* = 9) *C. coli* isolates were captured as multidrug resistant against three or more antimicrobial agents, viz, amoxicillin, streptomycin, tetracycline, erythromycin, ciprofloxacin, amoxicillin, norfloxacin and azithromycin. However, these are the most commonly used in poultry rearing in Bangladesh. As selective pressure is usually accountable for AMR development through indiscriminately use antibiotics as feed additives and growth promoters in poultry feed [13] that might have a direct association with the development of multidrug resistance *Campylobacter* in poultry [21]. However, government efforts have focused on the prudent use of antibiotics only for therapeutic purposes [57]. These resistant profiles of *Campylobacter* spp. as multidrug resistant are comparable to the observations of some other researchers in Bangladesh [13,19]. However, another study in Bangladesh detected as 86.36% *C. jejuni* and 100% *C. coli* isolates were found to be as multidrug resistant [18]. Furthermore, 49% and 42% isolates of *C. jejuni* and *C. coli*, respectively were confirmed as multidrug resistant and resistant to three or more antimicrobial agents in samples collected from poultry farms and LBMs of Bangladesh [21]. The spread of multidrug resistant bacteria can cause infections that are resistant to antibiotic treatment [58] and the transmission of residual antimicrobials through the food chain can trigger serious health problems, like gastrointestinal and neurological ailments, tissue damage, and hypersensitivity in animals and humans [59]. Therefore, the use of antimicrobials in food animal production should be minimized considering possible alternative options, like prebiotics, probiotics, and herbal extracts, for both prophylactic and therapeutic use in poultry production [60,61].

The study has some limitations as only single colony was used from each sample for subculture and finally molecular detection for species confirmation. This indicates that samples with more than one species of *Campylobacter* could not be captured under this study. Moreover, the samples were collected considerably from a limited number farms and did not to cover all poultry dominant districts of Bangladesh. In this regard, an in-depth longitudinal survey, including potential risk factors, is needed covering all poultry dominant districts with an aim to identify the potential drivers of *Campylobacter* spp. introduction and subsistence along the value chain (farm to table) as remedial options to be appropriate in the context of Bangladesh.

## 5. Conclusions

The study confirmed a substantial degree of *Campylobacter* contamination in a wide range of samples of a major poultry production system that signifies a huge public health concern. In addition, some isolates were defined as multidrug resistant, which creates extra burden for public health. In this regard, raising farmers’ awareness regarding good farm practices, including biosecurity measures, and the prudent use of antibiotics along with personal hygiene for poultry keepers need to be ensured through participatory training under a one health approach. These measures will pave the way for minimizing the burden of multidrug resistant poultry origin *Campylobacter* pathogens.

## Figures and Tables

**Table 1:** Five key biological control measures (bacteria) for safe broiler production.

Key Control Measures	Areas of Intervention
Protect poultry flock with good biosecurity practices	Perimeter fencing, netting at poultry sheds; poultry trucks & cages are cleaned & disinfected before entry into farm; footwear are cleaned and disinfected before entry and provision of quarantine facilities for suspected/possibly infected poultry.
Use safe production inputs that free of biological hazards	Commercial day-old-chicks or pullets reliable sources; veterinary health certificate for free from infectious diseases e.g., *Salmonella*, *Campylobacter* etc, and with certificate of origin, and water are free of biological hazards e.g., *Salmonella*, *Campylobacter*.
Apply good animal husbandry practices (part of good agriculture practices)	Vaccination against poultry diseases; age group separation e.g., buffer zones of >30 m, and practice all-in-all-out, appropriate stocking density (1.6 to 2.0 sq.ft./bird depending on size of broiler); use probiotics for preventing the pathogens; veterinary inspection before selling on good health status, and keep proper records and documentation of farm.
Practice good personal hygiene	Ensure workers’ health, wash hands after visiting toilet and handling live poultry or poultry waste, and before entering poultry sheds; use PPE (boots & gloves) when working in poultry sheds or cleaning poultry sheds; workers who are sick, with cuts or wounds or immunologically compromised should not work in the farm.
Practice good poultry waste management and environmental control (part of good agriculture practices)	Good poultry waste management (bury poultry waste with lime, compost), good pest control, Keep farm environment clean with good drainage in the farm and free from disused equipment and construction material in the farm.

**Table 2:** Details of primers used in this study.

Primers	Sequence (5′-3′)	Target Gene	Amplicon Size (bp)	PCR Condition (30 Cycle)			Reference
				Denaturation	Annealing	Extension	
16S9F 16S1540R	GAGTTTGATCCTGGCTC AAGGAGGTGATCCAGCC	16S rRNA	1530	94 °C, 30 s	47 °C, 30 s	72 °C, 90 s	[26]
HIP400F HIP1134R	GAAGAGGGTTTGGGTGGTG AGCTAGCTTCGCATAATAACTTG	*hipO* gene	735	94 °C, 30 s	55 °C, 30 s	72 °C, 45 s	[27]
CjspAU2 CjspAR2	AGGACTTGAACCTACTTTTC AGGTGGAGTAGTTAAAAACC	*Cj cdtA*	631	94 °C, 30 s	53 °C, 30 s	72 °C, 30 s	[28]
CcspAU1 CcspAR1	ATTGCCAAGGCTAAAATCTC GATAAAGTCTCCAAAACTGC	*Cc cdtA*	329				
CfspAU1 CfspAR1	AACGACAAATGTAAGCACTC TATTTATGCAAGTCGTGCGA	*Cf cdtA*	489				

**Table 3:** Occurrence of *Campylobacter* spp. in broiler farms of 3 districts of Bangladesh (*N* = 352 samples).

District/Sub-District (Upazila)	No. of Sample ^a^	No. of Positive Samples	Occurrence (%)	95% CI	*p* Value (Pearson’s Chi-Squared Test)
**District**					
Gazipur	264	70	26.5	21.3–32.3	0.76
Tangail	44	13	29.5	16.8–45.2	
Dhaka	44	10	22.7	11.5–37.8	
**Sub-District (Upazila)**					
Sadar, Gazipur	88	18	20.4	12.6–30.4	0.014
Sreepur, Gazipur	88	17	19.3	11.7–29.1	
Kapasia, Gazipur	88	35	39.8	29.4–50.8	
Sadar, Tangail	44	13	29.6	16.8–45.2	
Savar, Dhaka	44	10	22.7	11.4–37.8	
**Overall**	352	93	26.4	21.9–31.3	

^a^ Samples included cloacal swab, feed, drinking water, attendants’ hand rinse water, and whole carcasses.

**Table 4:** Occurrence of *Campylobacter* spp. in different types samples among two groups of broiler farms (good practice farms versus conventional farms).

Sample Type/Parameters	Farm Type (*n*)	No. of Positive Samples	Occurrence (%)	95% Confidence Interval	*p* Value (Pearson’s Chi-Squared Test)
Cloacal swab	Conventional farms (*n* = 64)	31	48.4	35.7–61.3	0.001
	Good practice farms (*n* = 64)	13	20.3	11.3–32.2	
	Both farms (*n* = 128)	44	34.4	26.2–43.3	
Feed	Conventional farms (*n* = 32)	8	25	11.5–43.4	0.35
	Good practice farms (*n* = 32)	5	15.6	5.3–32.8	
	Both farms (*n* = 64)	13	20.3	11.3–32.2	
Drinking water for poultry	Conventional farms (*n* = 32)	8	25	11.5–43.4	0.20
	Good practice farms (*n* = 32)	4	12.5	3.5–29	
	Both farms (*n* = 64)	12	18.8	10.1–30.5	
Attendants’ hand rinsed	Conventional farms (*n* = 32)	8	25	11.5–43.4	0.20
	Good practice farms (*n* = 32)	4	12.5	3.5–29	
	Both farms (*n* = 64)	12	18.8	10.1–30.5	
Whole carcass	Conventional farms (*n* = 16)	9	56.3	29.9–80.2	0.02
	Good practice farms (*n* = 16)	3	18.8	4–45.6	
	Both farms (*n* = 32)	12	37.50	21.1–56.3	
All samples	Conventional farms (*n* = 176)	64	36.4	29.3–43.9	0.000
	Good practice farms (*n* = 176)	29	16.5	11.3–22.8	
	Both farms (*N* = 352)	93	26.4	21.9–31.3	

**Table 5:** Distribution of antimicrobial resistance pattern among the isolated *C. jejuni* (*n* = 63) and *C. coli* (*n* = 30) strains from broiler farms.

Resistance against Antimicrobials	Resistance Patterns	*C. jejuni* (*n* = 63)		*C. coli* (*n* = 30)	
		No. (%) of Strains	Subtotal [No. (%)]	No. (%) of Strains	Subtotal [No. (%)]
**Against One to Two Antimicrobial Agents**					
Against one	AMX	5 (6.4)	32 (50.8)	3 (10)	17 (56.7)
	E	5 (7.9)		4 (13.3)	
	AZM	5 (7.9)		3 (10)	
	S	5 (7.9)		1 (3.3)	
	E	4 (6.4)		3 (10)	
	GEN	4 (6.4)		2 (6.7)	
	NOR	5 (7.9)		1 (3.3)	
Against two	AMX-TE	4 (6.4)	9 (14.3)	2 (6.7)	4 (13.4)
	AMX-S	5 (7.9)		2 (6.7)	
**Against Three or More Antimicrobial Agents**					
Against three	AMX-S-TE	5 (7.9)	12 (19)	2 (6.7)	5 (16.7)
	E-S-CIP	7 (11.1)		3 (10)	
Against four	AMX-NOR-AZM-TE	10 (15.9)	10 (15.9)	4 (13.3)	4 (13.3)
Total against three or more antimicrobials		22 (34.9)		9 (30)	

## Supplementary Materials

Supplementary Materials could be found at https://www.mdpi.com/2076-2607/8/11/1778/s1. The data that support the findings of this study are available at online. Table S1. List of the farms (good practice and conventional farms) from 5 sub-districts of 3 districts of Bangladesh. Sample is presented under each category as pooled sample of three subsamples from each farms. Equal number of samples (*n* = 176) were collected from each category of farms, Table S2. Sample collection checklist and Figure S1. 16S rRNA gene-based PCR to identify *Campylobacter* genus (a), *hipO* gene-based PCR to identify *C. jejuni* (b), and *cdtA* gene-based multiplex PCR assay to identify *C. coli* (c). In all figures, lanes: 1 and 8, 100 bp DNA ladder (Promega, Madison, WI, USA); lanes 2–5, representative positive isolates; lane 6, positive control (*C. jejuni* ATCC 33560 in picture a and b, and *C. coli* ATCC 33559 in picture c); and lane 7, negative control (*Escherichia coli* ATCC 25922).
