# Supplementary material for: Occurrence of Campylobacter spp. in Selected Small Scale Commercial Broiler Farms of Bangladesh Related to Good Farm Practices
Source: Microorganisms. 2020 Nov 13;8(11):1778. doi: 10.3390/microorganisms8111778 (PMC7709009; doi:10.3390/microorganisms8111778)
Supplement: Supplementary file 1 [file microorganisms-08-01778-s001.zip › proof -supp/Supplementary Table S1 appendix .docx]

**Supplementary Table S1 appendix: List of the farms (good practice and conventional farms) from 5 sub-districts of 3 districts of Bangladesh. Sample is presented under each category as pooled sample of three subsamples from each farms. Equal number of samples (n=176) were collected from each category of farms**

| **Farm ID** | **Sub-districts**  **(Upazila)** | **District** | **Number of birds** | **Age**  **(days)** | **Farm category** | **Cloacal swab** | **Whole carcass** | **Water** | **Hand rinsed water** | **Feed** | **Collection date** |
| --- | --- | --- | --- | --- | --- | --- | --- | --- | --- | --- | --- |
| 2102031 | Gazipur Sadar | Gazipur | 1500 | 37 | Good practice farm | 4 | 1 | 2 | 2 | 2 | 16-08-2017 |
| 21020101 | Gazipur Sadar | Gazipur | 2800 | 41 | Good practice farm | 4 | 1 | 2 | 2 | 2 | 16-08-2017 |
| 21020111 | Gazipur Sadar | Gazipur | 2150 | 36 | Good practice farm | 4 | 1 | 2 | 2 | 2 | 16-08-2017 |
| 21020122 | Gazipur Sadar | Gazipur | 2360 | 43 | Good practice farm | 4 | 1 | 2 | 2 | 2 | 16-08-2017 |
| 21020128 | Gazipur Sadar | Gazipur | 3120 | 39 | Good practice farm | 4 | 1 | 2 | 2 | 2 | 16-08-2017 |
| 2NPI-1 | Gazipur Sadar | Gazipur | 2000 | 37 | Conventional farm | 4 | 1 | 2 | 2 | 2 | 13-09-2017 |
| 2NPI-2 | Gazipur Sadar | Gazipur | 1900 | 35 | Conventional farm | 4 | 1 | 2 | 2 | 2 | 13-09-2017 |
| 2NPI-3 | Gazipur Sadar | Gazipur | 2450 | 34 | Conventional farm | 4 | 1 | 2 | 2 | 2 | 13-09-2017 |
| 2NPI-4 | Gazipur Sadar | Gazipur | 2300 | 42 | Conventional farm | 4 | 1 | 2 | 2 | 2 | 13-09-2017 |
| 2NPI-5 | Gazipur Sadar | Gazipur | 1840 | 35 | Conventional farm | 4 | 1 | 2 | 2 | 2 | 13-09-2017 |
| 31030116 | Tangail Sadar | Tangail | 2320 | 39 | Good practice farm | 4 | 1 | 2 | 2 | 2 | 26-09-2017 |
| 31030127 | Tangail Sadar | Tangail | 1600 | 42 | Good practice farm | 4 | 1 | 2 | 2 | 2 | 26-09-2017 |
| 3NPI-1 | Tangail Sadar | Tangail | 2200 | 41 | Conventional farm | 4 | 1 | 2 | 2 | 2 | 26-09-2017 |
| 3NPI-2 | Tangail Sadar | Tangail | 2700 | 40 | Conventional farm | 4 | 1 | 2 | 2 | 2 | 26-09-2017 |
| 21020217 | Kapasia | Gazipur | 1100 | 33 | Good practice farm | 4 | 1 | 2 | 2 | 2 | 5/10/2017 |
| 21020218 | Kapasia | Gazipur | 2900 | 44 | Good practice farm | 4 | 1 | 2 | 2 | 2 | 5/10/2017 |
| 2NPI-1 | Kapasia | Gazipur | 1200 | 40 | Conventional farm | 4 | 1 | 2 | 2 | 2 | 5/10/2017 |
| 2NPI-2 | Kapasia | Gazipur | 1900 | 42 | Conventional farm | 4 | 1 | 2 | 2 | 2 | 5/10/2017 |
| 21020301 | Sreepur | Gazipur | 2310 | 39 | Good practice farm | 4 | 1 | 2 | 2 | 2 | 11/10/2017 |
| 21020302 | Sreepur | Gazipur | 3100 | 37 | Good practice farm | 4 | 1 | 2 | 2 | 2 | 11/10/2017 |
| 21020304 | Sreepur | Gazipur | 1870 | 34 | Good practice farm | 4 | 1 | 2 | 2 | 2 | 11/10/2017 |
| 21020311 | Sreepur | Gazipur | 3100 | 38 | Good practice farm | 4 | 1 | 2 | 2 | 2 | 11/10/2017 |
| 21020315 | Sreepur | Gazipur | 3400 | 36 | Good practice farm | 4 | 1 | 2 | 2 | 2 | 11/10/2017 |
| 2NPI-1 | Sreepur | Gazipur | 2450 | 43 | Conventional farm | 4 | 1 | 2 | 2 | 2 | 19-10-2017 |
| 2NPI-2 | Sreepur | Gazipur | 3100 | 40 | Conventional farm | 4 | 1 | 2 | 2 | 2 | 19-10-2017 |
| 2NPI-3 | Sreepur | Gazipur | 2700 | 38 | Conventional farm | 4 | 1 | 2 | 2 | 2 | 19-10-2017 |
| 2NPI-4 | Sreepur | Gazipur | 2350 | 35 | Conventional farm | 4 | 1 | 2 | 2 | 2 | 19-10-2017 |
| 2NPI-5 | Sreepur | Gazipur | 2500 | 39 | Conventional farm | 4 | 1 | 2 | 2 | 2 | 19-10-2017 |
| 11010103 | Savar | Dhaka | 3400 | 36 | Good practice farm | 4 | 1 | 2 | 2 | 2 | 28-10-2017 |
| 11010107 | Savar | Dhaka | 3800 | 35 | Good practice farm | 4 | 1 | 2 | 2 | 2 | 28-10-2017 |
| 1NPI-1 | Savar | Dhaka | 2900 | 40 | Conventional farm | 4 | 1 | 2 | 2 | 2 | 28-10-2017 |
| 1NPI-2 | Savar | Dhaka | 3100 | 41 | Conventional farm | 4 | 1 | 2 | 2 | 2 | 28-10-2017 |
