# Supplementary material for: Occurrence of Campylobacter spp. in Selected Small Scale Commercial Broiler Farms of Bangladesh Related to Good Farm Practices
Source: Microorganisms. 2020 Nov 13;8(11):1778. doi: 10.3390/microorganisms8111778 (PMC7709009; doi:10.3390/microorganisms8111778)
Supplement: Supplementary file 1 [file microorganisms-08-01778-s001.zip › proof -supp/Supplementary Table S2 appendix .docx]

**Supplementary Table S2 appendix: Sample collection checklist**

**Name of the project: Occurrence of *Campylobacter* in selected small scale commercial broiler farms of Bangladesh related to good farm practices**

**Sample collection check list ID # ……..**

Farm code: ……………..

**Geo-spatial location:**

Northing…………………… Easting………………………

Location of farm:

Village:…………………..,Sub-district (Upazila): ………. , District: …………

Date of DOC received: ………….

Source of DOC (hatchery name): ………..

Source of Feed (feed mill): …………………… Date of arrival of feed at farm…/……/…….

Expected date of poultry sale: ………………………….

Any medicine/ chemicals were given to the flock while sampling? : Y / N

If yes: Please mention the name of medicine and dose: ……………………..

**Sample ID#**

| **Sample type (pooled)** | **Sample ID** | **Remarks** |
| --- | --- | --- |
| Feed |  |  |
| Water |  |  |
| Cloacal swab |  |  |
| Whole carcass |  |  |
| Hand rinse water of farm attendant |  |  |

| **Signature ……………………**  **(Research assistant/fellow)** | **Signature ………………………………..**  **(Research Supervisor)** |
| --- | --- |
