# Supplementary figures and images for: Occurrence of Campylobacter spp. in Selected Small Scale Commercial Broiler Farms of Bangladesh Related to Good Farm Practices
Source: Microorganisms. 2020 Nov 13;8(11):1778. doi: 10.3390/microorganisms8111778 (PMC7709009; doi:10.3390/microorganisms8111778)

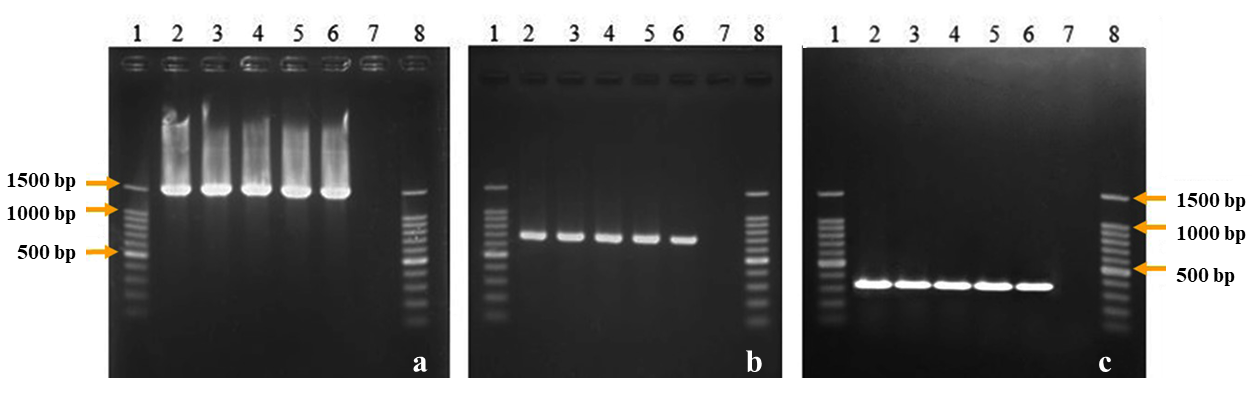

Supplement: Supplementary file 1 [file microorganisms-08-01778-s001.zip › proof -supp/Supplementary Figure S1 appendix.tif]
